# Human platelets support Semliki Forest virus replication

**DOI:** 10.1186/s13104-025-07470-2

**Published:** 2025-09-30

**Authors:** Cameron J. Stockwell, Matthew S. Hindle

**Affiliations:** Centre for Biomedical Science Research, School of Health, Leeds Beckett University, Leeds, LS1 3HE UK

**Keywords:** Blood platelets, Semliki Forest virus, Viral replication

## Abstract

**Objective:**

Platelets are blood cells which are critical for effective haemostasis and thrombosis. Beyond these classic functions of platelets, a range of roles for them in infectious diseases have also emerged. However little is currently understood about platelet-virus interactions. This study was performed to assess if human platelets are permissive to, and support replication of Semliki Forest virus (SFV), a commonly used model alphavirus.

**Results:**

Uptake of the virus by washed human platelets was demonstrated with fluorescent flow cytometry using eGFP fused SFV. To explore if uptake of virus leads to transcriptional replication, RT-qPCR for SFV nsP4 was performed on platelets infected with SFV. This showed a significant increase in nsP4 RNA which increased further over time. This is the first demonstration of SFV RNA increasing within platelets and suggests that platelets may be able to support viral replication, however further work is required to clarify if this leads to production of functional virions.

## Introduction

Platelets are among the most numerous cells in the blood and play a critical role in haemostasis and thrombosis [1]. It is also recognised that they play an important role in both sterile (i.e., non-infectious) and pathogenic (i.e., infectious) disease [2]. The roles of platelets in various viral conditions has been previously reviewed in the context of various viruses including influenza A, severe acute respiratory syndrome coronavirus 2, human immunodeficiency virus and dengue virus (DENV) [3, 4]. In addition to this platelets have been shown to express functional toll-like receptors (TLRs) capable of recognising viral material, which may mediate platelet-virus immune interactions [5]. This activity may in part underpin the inflammatory activity of platelets in viral haemorrhagic fevers such as DENV infection [6].

Semliki Forest virus (SFV) is an alphavirus which has been commonly used as a model of positive single-strand RNA (ssRNA) viral entry, replication, and pathogenicity over the last 50-years [7]. Here we have infected human platelets with SFV to measure if, (i) platelets can internalise SFV and, (ii) if SFV shows transcriptional activity in platelets.

## Main text

### Methods

#### Materials

 BD Vacutainer ACD-A Tubes (366645) were from BD Biosciences. Prostaglandin I_2_ (sodium salt) (61849-14-7) was from Cayman Chemical. Formaldehyde 16% (28908) was from Thermo Scientific. CD42b-PE/Cy7 (25-0429-42) and CD45-PE (12-0459-42) were from Invitrogen. Direct-zol RNA MiniPrep Plus (R2072) was from Zymo. Reverse transcription system (A3500) was from Promega. Itaq Universal SYBR^®^ Green Master mix (1725120) was from Bio-Rad. All other reagents where not specified were from Sigma-Aldrich.

#### Platelet isolation

 Blood was drawn from self-reported healthy adult donors, who provided informed consent using 21 g butterfly needles into ACD-A vacutainers. Platelet isolation was performed as previously described [8]. In brief, to isolate washed platelets (WP), anticoagulated blood was centrifuged at 100*g* for 20 min to isolate platelet-rich plasma (PRP). PRP was removed and treated with prostacyclin (PGI_2_) (200 nM) and then centrifuged at 800*g* for 12 min to pellet platelets. The platelet pellet was resuspended in a wash buffer of 90% (v/v) modified Tyrode’s buffer (0.5 mM MgCl_2_, 0.55 mM NaH_2_PO_4_, 2.7 mM KCl, 5 mM HEPES, 5.6 mM glucose, 7 mM NaHCO_3_, 150 mM NaCl, pH 7.4) and 10% (v/v) ACD (2.9 mM citric acid, 29.9 mM sodium citrate, 72.6 mM NaCl, 113.8 mM glucose, pH 6.4) then treated with PGI_2_ (200 nM) and centrifuged at 800*g* for 12 min to pellet platelets. The final pellet was resuspended in pre-warmed (37 °C) modified Tyrode’s buffer. Platelets were counted using 96-well microtitre plate by light absorbance at 405 nm, absorbance was used to calculate count against a pre-calibrated absorbance-count standard curve using methods similar to those described elsewhere [9], and this was used to adjust the final count to 5 × 10^8^ platelets/mL.

#### Virus production

 HeLa cells were grown in minimum essential medium Eagle (EMEM, M4655; Gibco) complete with Glutamax, foetal bovine serum (5% v/v, Gibco) and non-essential amino acids (1% v/v, Gibco). For virus studies, an infectious SFV clone derived from pSP6-SFV4 was used [10]. For fluorescent studies a modified SFV4 incorporating a uncleavable fusion eGFP to the C-terminal of the viral non-structural protein 3 (nsP3) was used (SFV nsP3-eGFP) [11]. To generate virus, confluent monolayers (80%) of HeLa were infected with SFV and grown for 24 h prior to harvesting by centrifugation at 1000*g*, 10 min, subsequently virus titre was determined by plaque assay on HeLa monolayer.

#### Flow cytometry

 For virus flow cytometry, 5 × 10^8^ washed platelets/mL were incubated with SFV nsP3-eGFP (multiplicity of infection (MOI) = 10) or an equivalent volume of low serum EMEM (mock) for 3.5 h followed by three centrifugal washes at 800*g* for 10 min. These treated and washed WP were stained in modified Tyrode’s buffer with anti-CD42b-PE/Cy7 in the dark for 20 min. For leukocyte contamination flow cytometry, 5 × 10^7^ washed platelets/mL or sodium citrated whole blood (1/10 dilution) were incubated with CD45-PE for 20 min. Samples were fixed with a 10x volume of PFA/PBS (1% v/v) prior to analysis on a BD C6 Accuri 2-laser/4-detector flow cytometer. Data analysis was performed using Floreada (https://floreada.io).

#### RNA isolation and RT-qPCR

 Following 1, or 4 h of infection with SFV (MOI = 10) or mock treatment, washed platelets were pelleted at 800*g* for 5 min and supernatants removed prior to pellet lysis with RNAzol^®^ RT. To isolate total RNA, a ZYMO Direct-zol RNA isolation kit was used following the manufacturer’s instructions, inclusive of a 30 min DNAse digestion. Primers were from Sigma-Aldrich and diluted as recommended with nuclease free water. Primers were designed for the following genes; *nsP4*:F (5’-CCG CCC CGT GTA CTC CCC TA-3’) and *nsP4*:R*-* (5’-AGC TTC GCC GGG CAG AAT GT-3’); *GAPDH*:F (5’-TTC ACC ACC ATG GAG AAG GC-3’) and *GAPDH*:R (5’-GGC ATG GAC TGT GGT CAG A-3’); *CD45*:F (5’-CTT CAG TGG TCC CAT TGT GGT G-3’) and *CD45*:R (5’-CCA CTT TGT TCT CGG CTT CCA G-3’). To produce cDNA, a Promega reverse transcription system was used and subsequent qPCR was performed using a Bio-rad Itaq Universal SYBR^®^ Green Supermix. Gene expression was determined using a Bio-rad CFX96 Touch Real-Time PCR Detection System with fluorescence filtered for FAM/SYBR^®^ only.

## Results

**Fig. 1:**
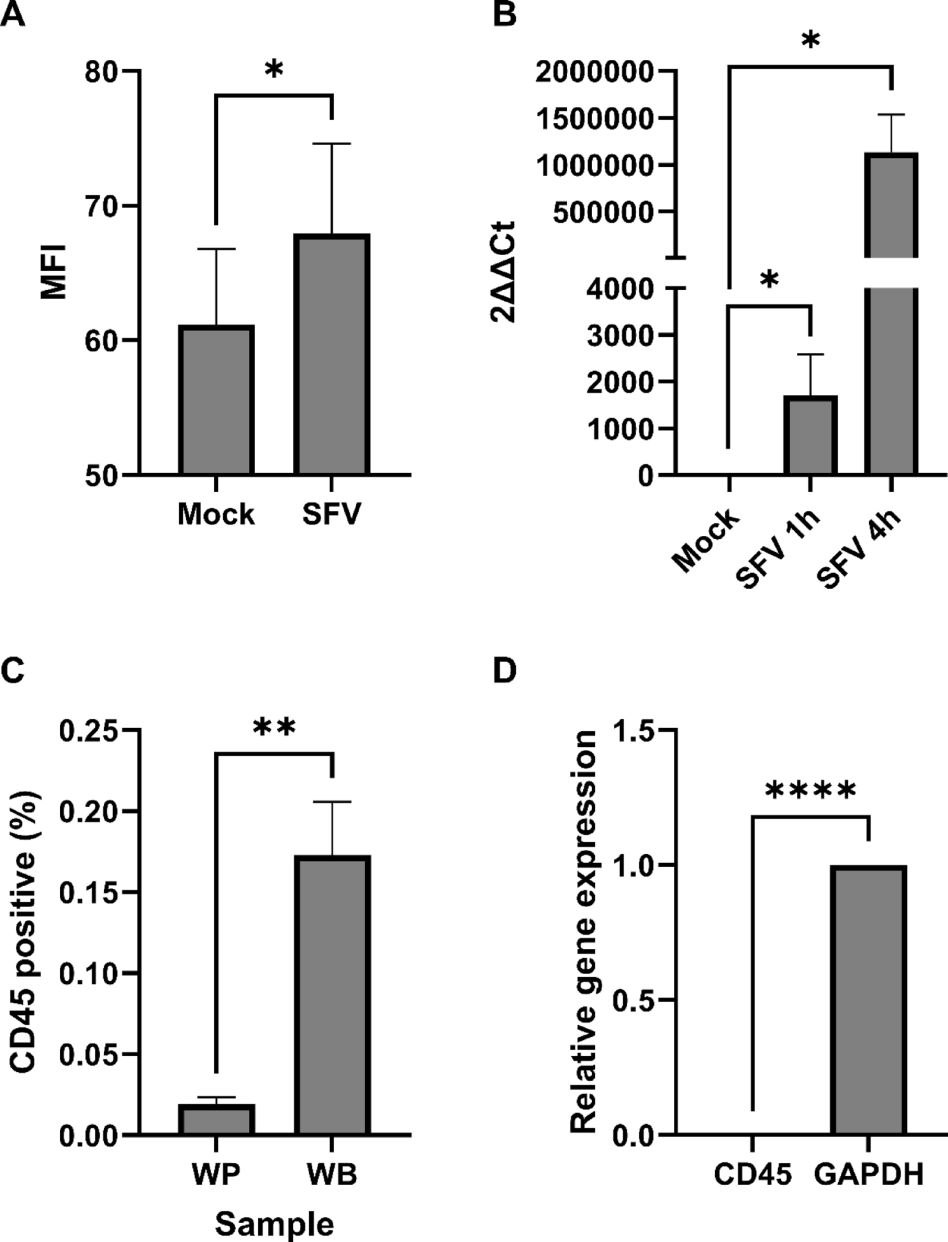
Semliki Forest virus is taken up and replicates in washed human platelets. **A** Washed human platelets (5 × 10^8^/mL) incubated with SFV nsP3-eGFP (MOI = 10, 4 h, 37 °C), washed and then analysed by flow cytometry in FL1. *n* = 3, paired 1-tailed T-test. **B** Washed human platelets (5 × 10^8^/mL) incubated with SFV (MOI = 10, 1–4 h, 37 °C), washed, RNA isolated and analysed by RT-qPCR for expression of SFV nsP4. *n* = 3 paired 1-tailed T-test. **C** Washed human platelets or whole blood stained with CD45-PE and analysed by flow cytometry for positive events in FL2. *n* = 3 paired 1-tailed T-test. **D** RNA isolated from washed human platelets and analysed by RT-qPCR for expression of CD45. *n* = 3 paired 1-tailed T-test

In the first instance, we considered if platelets can internalise SFV. We initiated the infection of washed human platelets with SFV nsP3-eGFP (MOI = 10, 4 h, 37 °C). MOI = 10 was used as it has been used in other platelet virology studies and supports robust infection for proof of principle studies [12]. Infected, or mock infected platelets were washed and subsequently analysed for eGFP signal by flow cytometry. After incubation there was a small but significant increase (*P* = 0.0310) in SFV nsP3-eGFP signal over mock treated (Fig. 1A). This represents uptake, as post viral incubation the samples were washed to remove unbound and/or surface bound virus. These experiments indicate that platelets do appear to take up SFV, however this did not explore if platelets can support SFV transcriptional activity. To assess this, washed platelets were infected with SFV (MOI = 10) and incubated for either 1–4 h (37 °C). RNA was isolated and SFV nsP4 expression was quantified using RT-qPCR. At both 1 h (*P* = 0.0395) and 4 h (*P* = 0.0198) incubation, SFV nsP4 was significantly upregulated in washed human platelets (Fig. 1B) compared to mock treatment and increased over time. These data indicate that not only are human platelets permissive to SFV, but SFV also undergoes some replicative activity within platelets.

To confirm that the increased detection of gene expression for SFV nsP4 was dependent on platelets and not other contaminating blood cells, purity of the washed platelet isolate was confirmed. First by flow cytometry, which demonstrated there was no clear CD45 + population present in washed platelets (0.019%±0.003, *≤* 1/5,263 CD45 + cells per platelet), in comparison, CD45 + cells were detectable at the expected levels in whole blood (0.173%±0.027, Fig. 1C). Secondly, CD45 expression was measured by RT-qPCR from lysed washed platelet suspensions. Relative gene expression of CD45 was significantly below (3.5 × 10^− 5^, p = < 0.0001) the expression of the housekeeping transcript GAPDH indicating minimal contamination of CD45 positive cells (Fig. 1D). Validating that the platelet suspensions were pure confirms the likelihood that SFV undergoes *bona fide* transcriptional activity within washed platelets.

## Discussion

Typically, the roles of platelets in viral disease are assumed to be passive, haemostatic, or playing a role in immune response, a component of this concept is the expectation that as non-nucleated cells platelets have limited capacity to impact on viral lifecycles. As platelets lack nuclei it is unlikely that they would be able to support replication of some classes of viruses (i.e., DNA viruses), although they remain able to interact and modulate immunity when stimulated by them [13]. However, platelets may be able to support positive single-strand viral replication [14], where the viral genome can be directly transcribed following virus entry. This is supported by the fact that platelets, while not nucleated cells, are described as carrying diverse pools of RNA [15]. Indeed, platelets have been shown to have translational activity and are able to splice and produce new proteins from their own mRNA stores [16]. It is likely this capacity which underpins the capacity of platelets to support SFV replication. The data published here is the first indicating that SFV, or indeed any alphavirus, may replicate within platelets. Similar studies by others on DENV–platelet interactions have shown that platelets can support DENV replication [17]. Critically we confirm in these experiments that this is platelet-specific, as fluorescent uptake is gated specifically on platelets, and for RNA replication studies there is minimal contamination by white blood cells.

Early SFV studies indicated that SFV can produce new viruses in nucleated cells within 3 h of infection [18], with later studies confirming that significant amounts of virus are notable from 2 h [19, 20]. The nsP3-eGFP SFV is considered to lag behind non-eGFP SFV by approximately 1 h within the first 5 h of infection [11]. The significant increase in eGFP signal we observe at the timepoint of 3.5 h falls within these timepoints and is likely indicative of uptake of SFV and may also represent early replicative activity, longer incubations to allow further viral replication may demonstrate further increases in SFV nsP3 eGFP signal.

As SFV is an alphavirus it is a particularly relevant model to Chikungunya virus (CHIKV). Given the increasingly global burden of ssRNA alphavirus virus infection, with close to 19 million CHIKV cases between 2011 and 2020 [21], this remains a critically important and under-researched field. CHIKV is considered a virus of concern, and since its re-emergence it has caused several large-outbreaks and carries a substantial mortality burden [22]. Other studies have indicated that platelet concentrates are able to maintain infective CHIKV for up to 5-days [23]. Taken together with our findings, this indicates platelets may play a role in CHIKV infection. Overall, the observations made here require further validation and exploration, preferentially translating findings made with the model virus SFV into more relevant human pathogens such as CHIKV to understanding the underlying molecular mechanisms and subsequent translational impact of of platelet-virus interactions.

## Limitations

This study has been able to identify (i) uptake of SFV by platelets and, (ii) transcriptional activity of SFV in platelets. While these are both novel findings, this data does not assess the critical question of whether platelets are producing functional viruses. Initial studies in DENV suggested that similar approaches lead to functional virion production [17], however subsequent studies on DENV suggested that while uptake and replicative activity are observed this does not lead to functional virus release [12]. To explore SFV-platelet interactions further, infected platelets should undergo viral harvest, and plaque forming unit assays carried out to determine if the uptake and transcriptional activity described here leads to production of platelet derived functional virus. Furthermore, the findings made here using model viruses such as SFV should be translated to other alphaviruses which are considered to have a significantly greater human health burden such as CHIKV. Ultimately detailed molecular studies should be considered to elucidate pathways controlling the uptake, replication and morphogenesis of alphaviruses in platelets.

## Data Availability

The datasets used in this study are available from the corresponding author on reasonable request.

## Abbreviations

CHIKV: Chikungunya virus
eGFP: Enhanced green fluorescent protein
h: Hour
Min: Minute
MOI: Multiplicity of infection
nsP4: Non-structural protein 4
PCR: Polymerase chain reaction
PGI_2_: Prostacyclin
PRP: Platelet-rich plasma
RT-q: Reverse transcription quantitative
SFV: Semliki Forest virus
ssRNA: Single-strand RNA
TLR: Toll-like receptor
v/v: Volume/volume
WP: Washed platelets
